# Abnormal susceptibility to distracters hinders perception in early stage Parkinson's disease: a controlled study

**DOI:** 10.1186/1471-2377-6-43

**Published:** 2006-12-11

**Authors:** Jan Berend Deijen, Diederick Stoffers, Henk W Berendse, Erik Ch Wolters, Jan Theeuwes

**Affiliations:** 1 Department of Clinical Neuropsychology, Vrije Universiteit, van der Boechorststraat 1, 1081 BT, Amsterdam, The Netherlands; 2 Institute for Clinical and Experimental Neurosciences, Department of Neurology, VU University Medical Center, P.O. Box 7057, 1007, MB, Amsterdam, The Netherlands; 3 Department of Cognitive Psychology, Vrije Universiteit, van der Boechorststraat 1, 1081 BT, Amsterdam, The Netherlands

## Abstract

**Background:**

One of the perceptual abnormalities observed in Parkinson's disease (PD) is a deficit in the suppression of reflexive saccades that are automatically triggered by the onset of a peripheral target. Impairment of substantia nigra function is thought to lead to this reduced ability to suppress reflexive saccades.

**Methods:**

The present study examined whether this perceptual deficit is also present in early stage PD when using hardly noticeable task-irrelevant stimuli. Eleven non-demented *de novo*, untreated PD patients (mean age 57 yr, range 44 – 70) participated in the study as well as 12 age-matched controls. Performance on an 'oculomotor capture' task, in which in half of the trials an irrelevant stimulus with sudden onset was added to the display, was compared between patients and controls. Analysis of variance (ANOVA) was performed with group (patients/controls) and age (< 61 yrs/≥ 61 yrs) as independent factors and type of trial (control/distracter) as repeated measurements factor. The factor sex was used as covariate.

**Results:**

With respect to Reaction Time (RT), a significant interaction between group and condition was found. RTs increased under the 'irrelevant stimulus' condition in both groups, the patients exhibiting a significantly larger increase in RTs than the control group. Also, a significant interaction effect between group and condition for number of correct responses was found. The number of correct responses was reduced in the onset distracter condition, the reduction being larger in the patients. In the patient group, contrary to the control group, a higher age was associated with fewer correct responses at baseline and in the onset distracter condition, suggesting that perceptual functions in PD are highly susceptible to the effects of ageing. The increased reaction times and larger number of incorrect responses of the PD patients in the onset distracter condition may be related to impairments of substantia nigra function and lower brain stem.

**Conclusion:**

The capture task seems to be a sensitive instrument to detect early perceptual deficits in PD. The magnitude of the observed deficits suggests that perceptual functions in early stage PD are so substantially impaired that this may interfere with daily activities.

## Background

Parkinson's disease (PD) is classically characterized by cardinal motor signs such as bradykinesia, muscular rigidity, tremor and postural instability. In addition to these motor symptoms, cognitive and perceptual changes are frequently reported in the literature. With respect to perceptual skills, changes in the various modalities of elementary sensory-perceptual functions (e.g., visual, olfactory, auditory and somatosensory) have been reported and may reflect impairment of either basic sensory systems per se or disturbances in higher order processes affecting these basic sensory systems [1]. With respect to visual perception, abnormalities in electroretinogram (ERG) and visual evoked potentials (VEP) point to changes in absolute sensitivity and in spatial contrast sensitivity [2,3]. The temporal sensitivity of PD patients seems to be altered, considering delayed ERGs and VEPs and changes in sensitivity to different temporal frequencies of visual stimuli [4,5].

A frequently used method for investigating perceptual processes in PD is the recording of eye movements. Abnormalities of saccade latencies and smooth pursuit gain in PD point to perceptual deficits [6,7]. With respect to saccades, it has been demonstrated that there are defects of predictive saccades [7,8], saccades to remembered target locations [9,10], and impaired ability to perform the anti-saccade task [11]. In addition, deficits in the suppression of reflexive saccades, i.e. saccades automatically triggered by the onset of a peripheral target, have been observed in PD [12]. There are neurophysiological indications that two pathways are involved in the generation of saccades, the parietal eye field (PEF)-superior colliculus (SC) pathway and the frontal pathway, consisting of the frontal eye field (FEF), the supplementary eye field (SEF) and the dorsolateral prefrontal cortex (DLPFC) [13]. The generation of purposeful saccades to a target largely depends on the frontal pathway, whereas reflexive saccades are thought to be dependent on the PEF-SC pathway [14].

Reflexive saccades, which involve the PEF and the SC of the midbrain, can be inhibited by the substantia nigra [13]. In addition, the DLPFC can inhibit unwanted reflexive saccades because of its involvement in decisional processes governing oculomotor behavior. The inhibition of reflexive saccades originating in the DLPFC is probably directly exerted on the SC [14]. The activation or inhibition of the visual grasp reflex within the SC is determined by competition between caudal and rostral collicular neurons. Neurons in the caudal region of the SC generate saccades while neurons in the rostral SC maintain the eyes anchored at fixation [15]. Pharmacological inactivation of these rostral neurons leads to disinhibition of reflexive saccades [16]. The extent to which an eccentric visual stimulus can trigger a visual grasp reflex is determined by the relative activity of neurons in the caudal and of those in the rostral regions of the SC. In addition to the involvement of the SC and substantia nigra in controlling saccades, the reticular formation appears to be especially important in organizing fast, saccadic eye movements that may be initiated by signals from the FEF or the SC, which sends excitatory fibers to neurons in the pontine reticular formation that produce saccadic eye movements [17].

As is stated above, the suppression of automatic, visually triggered saccades depends on the activity of rostral neurons in the SC which is likely influenced by the substantia nigra and the DLPFC. As the DLPFC is involved in one of several discrete basal ganglia-thalamocortical circuits, in turn it receives significant input from substantia nigra pars reticulata via the thalamus [18]. Thus, loss of dopaminergic input to the striatum, in particular the caudate nucleus, in PD may directly, or indirectly by the disruption of prefrontal basal ganglia-thalamocortical circuitry, lead to impairment of automatic response suppression. Indeed, a post-mortem neurochemical analysis by Kish et al. showed that, although the putamen is the most severely DA depleted structure in PD, dopamine levels in the rostral part of the caudate nucleus are also substantially depleted [19].

The evidence described above indicates that the control of reflexive saccades is impaired in PD. However, in most of the eye movement studies reflexive saccades are elicited by stimuli that are quite obvious and/or have some relevance for task performance. For instance, in the frequently used anti-saccade task the location of the stimulus *instructs *the subject that a saccade has to be made in the opposite direction. Thus, in the anti-saccade task the stimulus is in fact task-relevant [20]. In addition, predictive saccades or saccades to remembered targets are determined in response to obvious task stimuli [12]. Knowledge is still lacking regarding the extent to which patients with PD are unable to suppress reflexive saccades to stimuli that are truly task-irrelevant, i.e. to stimuli that are truly exogenous and of which observers typically are not aware. The aim of the present study was to find out whether a deficit in the control of reflexive saccades in PD still exists when hardly noticeable task-irrelevant stimuli are being used.

Typically, the measurement of eye movements can be problematic in patients with PD because of the requirement to hold the head as stable as possible. Therefore, in the present study we used a perceptual task to determine the suppression of reflexive saccades without the registration of eye movements. In this visual search task, also known as oculomotor capture task, subjects are required to make a voluntary, goal-directed saccade to discriminate a target letter appearing within a gray circle. In half of the trials, simultaneous with the presentation of the gray circle, a new, irrelevant stimulus suddenly appears somewhere in the display [20]. The requirement for this task is to discriminate the orientation of the target letter inside the gray circle and make a motor response with the index finger of the right or left hand. We expected that, in comparison with healthy controls, PD patients would exhibit longer reaction times on the regular part of this task and progressively longer reaction times under the condition of the appearance of an irrelevant distracter. If so, this would indicate that PD patients have a reduced capacity for inhibition of saccades to irrelevant stimuli. The present study was performed in early stage, untreated PD patients to exclude the possible influence of dopaminergic medication on perceptual parameters. Furthermore, the presence of perceptual deficits in early stage PD patients might explain subtle disturbances in the activities of daily living and, moreover, contribute to the development of sensitive diagnostic instruments to be used in the early detection of PD.

## Methods

### Patients

Eleven non-demented, early stage, untreated patients with idiopathic Parkinson's disease participated. Patients were selected from the outpatient clinic for movement disorders of the VU University Medical Center (VUMC). Inclusion criteria were an age between 40 and 77 years and a clinical diagnosis of PD according to the UK Parkinson's Disease Society Brain Bank criteria [21]. Unified Parkinsons's disease Rating scale (UPDRS) motor scores were obtained by a trained neurologist. Disease duration was estimated on the basis of the patients' subjective estimate of the time of occurrence of the first symptoms of Parkinson's disease. Twelve self-declared neurologically healthy subjects served as control. Groups were matched for age and education. Exclusion criteria for both groups were restricted mobility, head trauma, head tremor, and the use of psychoactive compounds. Education level was determined by means of the Dutch SOI (Standaard Onderwijs Indeling)-scale [22]. All subjects gave written informed consent to the research protocol, which was approved by the local medical ethical committee of the VUMC. Ethics review criteria conformed to the Helsinki declaration. Subject characteristics are listed in Table 1.

### Procedure

Subjects were examined individually in a dimly lit, quiet room. The test procedure as a whole took about 30 min. They were seated in an adjustable chair with their heads fixed in a head rest, placed at 0.9 m in front of a computer screen.

#### Capture task

In the present study, we used a visual search task in which subjects were required to make a voluntary, goal-directed saccade to a gray-singleton target. In half of the trials, simultaneous with the presentation of the gray-singleton target, an additional stimulus suddenly appeared somewhere in the display. Because the eyes would involuntarily move to the location of the irrelevant new stimulus, responses were expected to be slowed down by the appearance of the new stimulus. Typically, subjects were unaware that their eyes moved into the direction of the irrelevant distracter [20]. Even though the current task has some similarity to the antisaccade task, it should be noted that unlike the antisaccade task, in the current task the onset distracter was truly task-irrelevant. Indeed, in our task the target never appeared at the location of the onset distracter. Note that in the anti-saccade task the stimulus is relevant because its location *instructs *the participant that a saccade has to be made in the opposite direction. Hence, in the anti-saccade task the stimulus is in fact task-relevant. Thus, much more than in a antisaccade task, the onset distracter in the current task is a truly exogenous event that should be ignored [20].

#### Task specification

Initially, 6 gray circles (3.7° in diameter), each containing a small gray figure-eight premask, were equally spaced around an imaginary circle with a radius of 12.6°. After 1,000 ms 5 of the circles changed to red and at the same time the premasks inside the circles changed to letters randomly sampled without replacement from the set *S*, *E*, *H*, *P*, *F*, and *U*. Subjects were instructed to foveate the remaining gray circle and to determine whether the letter inside the gray target circle was a *c *(to which they responded by pressing a button with their right hand) or a reverse *c *(to which they responded by pressing a button with their left hand). In total, subjects performed 64 randomly mixed trials consisting of 32 trials in which no onset was present and 32 trials in which an abrupt onset appeared at the other side of the display relative to where the target was presented.

**Figure 1:**
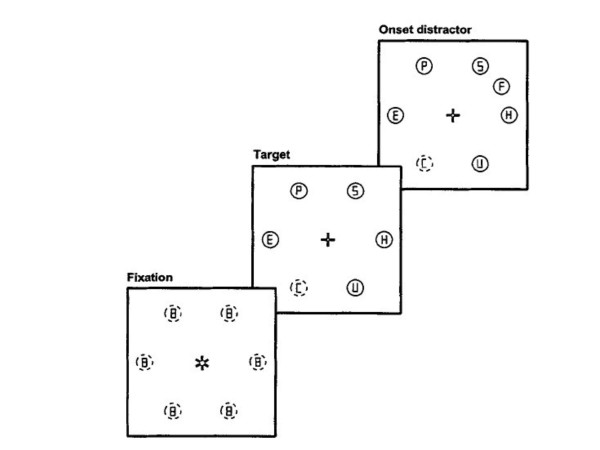
Graphic illustration of the capture task.

A graphic illustration of the task is shown in Fig. 1.

### Data analysis

The dependent variables used were the reaction time (RT) and the number of correct responses. As all variables were found to have a normal distribution, parametric tests could be performed. Statistical analysis comprised analyses of variance (ANOVA) with group (patients/controls) and age (< 61 yrs/≥ 61 yrs) as independent factors and condition (control/onset) as repeated measurements factor. Only RTs of trials with correct responses were included in the analyses. As the number of males and females was not similar in the patient and control groups, the factor sex was used as covariate. Data from one patient was not evaluated because of unreliable RTs, opposite to the expectation within the model (longer RTs for the control condition) and extremely deviating from the data of the other subjects. In addition, for the patient group the Pearson correlation coefficient of disease duration, scores for Hoehn and Yahr stage/UPDRS with reaction times and number of correct responses were calculated. Significance level was defined as *p *≤ .05 (two-tailed). Data were analysed using the SPSS version 11 software package (SPSS inc., Chicago, USA).

## Results

**Figure 2:**
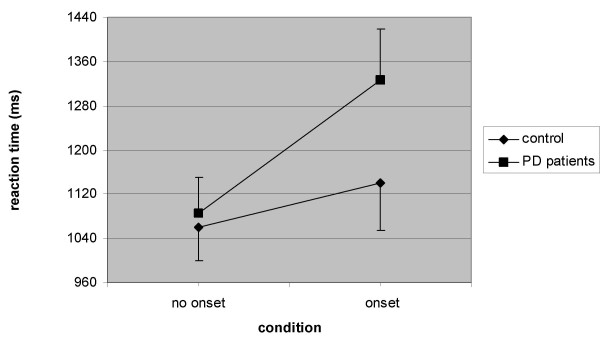
Mean RTs (± SEM) for onset distracter and control conditions in PD patients and controls.

With regard to RT, there was a main effect of the factor condition. RTs were significantly longer under the onset distracter condition (control 1073 ± 193 ms, onset 1230 ± 294 ms; *F *(1,18) = 31.97, *p *< 0.0001, η^2 ^= 0.64). With respect to RT, a main effect of group was not found. Further, there was a significant interaction effect between group and condition (*F *(1,18) = 4.78, *p *= 0.04, η^2 ^= 0.21). As is clear from Fig. 2, the RT's increased in the onset distracter condition for both groups, while the patients exhibited a relatively larger increase in RT's than the control group.

**Figure 3:**
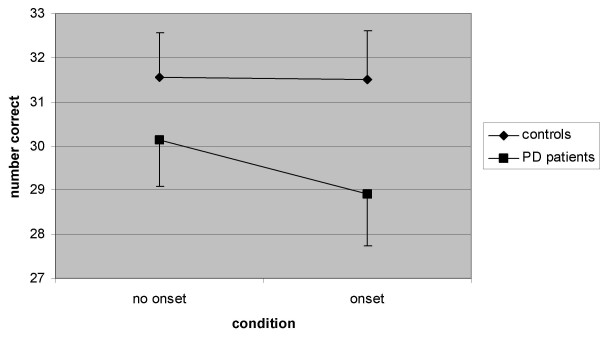
Mean number of correct responses (± SEM) for onset distracter and control conditions in PD patients and controls.

Regarding the number of correct responses, there was also a main effect of the factor condition (*F *(1,18) = 13.14, *p *= 0.002, η^2 ^= 0.42), the number of correct responses being smaller under the onset distracter condition (control 30.87 ± 3.37, onset 30.26 ± 3.80). Similar as for RT, no main effect of group was found concerning correct responses. In contrast, a significant interaction effect between group and condition was found (*F *(1,18) = 9.28, *p *= 0.007, η^2 ^= 0.34). Fig. 3 shows that the number of correct responses is reduced in the onset distracter condition, the reduction being larger in the patients.

For the number of correct responses, but not for RT, a significant interaction effect between group, age and condition was found (*F *(1,18) = 8.54, *p *= 0.009, η^2 ^= 0.32). ANOVAs separately performed for each group indicated that only in the PD group age interacted with condition (*F *(1,8) = 8.25, *p *= 0.02, η^2 ^= 0.51), indicating that the higher age group within the PD group made less correct responses in the control condition (age < 61: 31.17 ± 1.6, age ≥ 61 yrs: 28.4 ± 7.0) and even less in the onset condition (age < 61: 31.0 ± 2.4, age ≥ 61 yrs: 26.2 ± 6.7).

Finally, we performed a correlation analysis for the data obtained in the patient group. We computed these correlations to examine whether an impaired test performance in PD can be attributed to motor dysfunction and/or can be expected to become more impaired in the course of the disease. The number of correct responses and the RTs under both onset and control conditions were not correlated with Hoehn and Yahr stage, UPDRS scores or disease duration.

## Discussion

The present study was designed to examine whether patients in an early phase of PD have a reduced capacity to inhibit reflexive saccades to irrelevant stimuli. As the measurement of eye movements can be problematic in patients with PD a task was presented that enables to determine the effect of a distracter on reflexive eye movements without the registration of eye movements. When the appearance of an irrelevant distracter stimulus lengthens the reaction time in one person more than in the other, this would indicate that this person exhibits less inhibition of saccades elicited by an external cue. Obviously, it can be argued that the present oculomotor task not only measures eye movements but also other parameters concerning perception, information processing, movement response initiation and execution. However, in the first place we did not find differences between patients and controls for number of correct responses or RT in the no onset condition nor any main effects for group. This means that basically the performance of the PD patients on this task is similar to that of the controls. Secondly, as we did not observe any relationships between UPDRS scores of the patients and speed and quality of test performance, it does not seem likely that impaired test performance in the PD patients can be attributed to motor dysfunction. Thus, although other parameters than eye movements may be involved in the performance of this task, a reduced capacity for these perceptual and motor requirements should not be assumed to be present in these early PD patients. Thus, reduced performance in the onset distracter condition, the condition that distinguishes PD patients from controls, can mainly be attributed to a reduced inhibition of reflexive eye movements.

As indicated above, there are a number of different ways in which saccades can be used to foveate a target. In the most commonly used saccadic paradigm, which we also assume applicable in the oculomotor capture task, a saccade is triggered 'reflexively' to the onset of a peripheral target. Other paradigms require a more 'volitional' element in the saccade generation. For example, in the 'antisaccade' paradigm saccades have to be executed to a location opposite of that of the target. In the 'remembered' target paradigm a saccade is summoned to the location where a target was previously briefly presented. Frontal lesions produce impairments of anti-target and remembered target saccades, while reflexive saccades seem to be unimpaired [23]. Indeed, the generation of purposeful saccades to a target may depend on the frontal pathways, whereas the PEF-SC pathway is involved in the production of reflexive eye movements. A reflexive saccade, thus triggered by the PEF and generated by the SC, can be inhibited by the substantia nigra. Although the DLPFC may also be involved in inhibiting unwanted reflexive saccades, this may especially concern reflexive saccades one is aware of. Previous studies have shown that at least on a subset of trials subjects are not aware of the presence of an abrupt onset [20,24]. Because the abruptly appearing stimulus only briefly captures attention and the eye, it can be argued that the event does not reach awareness [20]. If indeed subjects are not aware of the irrelevant stimulus and the associated eye movements, involvement of the DLPFC in the present study is questionable. However, dopamine depletion in rats and monkeys has been found to increase the inhibitory output of the basal ganglia to the SC, which may lead to the suppression of saccadic eye movements [25,26]. As also in untreated patients with PD the loss of dopaminergic neurons in the substantia nigra may be expected to result in increased inhibition of the colliculus we would even expect a reduction of reflexive saccades to irrelevant stimuli. Therefore, the DLPFC may yet be involved in the observed deficit in suppression of reflexive saccades, in such a way that loss of dopaminergic input to the striatum leads to reduced DLPFC activity by disruption of prefrontal basal ganglia-thalamocortical circuitry.

Interestingly, within the patient group and not in the control group, a higher age was associated with fewer correct responses in the control condition and even less in the onset distracter condition. The absence of an effect of age in the control group is in line with the observation that young and old adults' eye movements to task-irrelevant stimuli are equivalently influenced when awareness of the task-irrelevant stimuli is low [27]. Similarly, detrimental effects of age on task performance have been found in PD patients but not in controls when using the Corsi blocks task, which measures visuo-spatial short term-memory. This would suggest that PD patients are more susceptible to cognitive ageing than healthy elderly subjects [28]. In another study, free recall on Rey's auditory verbal learning test (AVLT) in elderly PD patients was relatively more impaired than that in elderly controls compared to respectively younger patients or controls [29]. Furthermore, axial motor impairment in PD has been found to result from the combined effect of the disease and the aging process [30]. Taken together, these findings suggest that PD patients may be abnormally susceptible to the effects of aging on perceptual processes, memory performance and motor signs.

The capture task used in this study was expected to be a sensitive instrument to assess eye movement abnormalities in *de novo *PD patients. We expected that PD patients would show a larger increase of RTs and/or number of incorrect responses in the distracter condition relative to the control condition than normal subjects. Indeed, our patient group showed substantially increased reaction times and number of incorrect responses under the distracter condition as compared to healthy controls. Since the PD patients in the present study were at a very early disease stage (mean Hoehn and Yahr stage 1.4; mean disease duration 2.3 years) and were not using dopaminergic medication, it is likely that the subnormal performance on the capture task is related to the loss of dopaminergic neurons in the substantia nigra. The effect sizes for both dependent variables are above 0.14 which means that they can considered large. Thus, from a statistical point of view the abnormal performance on the capture task in PD patients is substantial. Therefore, from a clinical point of view impaired performance on this task may point to a reduced capacity of inhibiting attention to irrelevant stimuli also in daily life. It may well be true that minor alterations in basic sensory processes in early stage PD patients negatively affect attentional requirements of complex daily situations.

It is important to note that PD-related lesions in lower brain stem and anterior olfactory structures have been found that are present prior to involvement of the substantia nigra. Results of a semi-quantitative study of 30 autopsy cases with incidental Lewy body pathology indicate that PD in the brain may commence in non-catecholaminergic neurons of the dorsal glossopharyngeus-vagus complex, in projection neurons of the intermediate reticular zone, and in specific nerve cell types of the gain setting system (coeruleus-subcoeruleus complex, caudal raphe nuclei, gigantocellular reticular nucleus), olfactory bulb, olfactory tract, and/or anterior olfactory nucleus [31]. As stated in the introduction, in addition to the SC and the substantia nigra, the pontine reticular formation is also involved in the generation of eye movements. Metyrosine-induced catecholamine depletion in the human brain stem has been found to produce irrepressible saccadic eye movements [32]. In addition to increased saccade latencies impairment of the reticular formation may thus lead to a deficit in the suppression of eye movements. Therefore, the increased reaction times and higher number of incorrect responses of the PD patients in the onset distracter condition may also be related to a functional impairment of the reticular formation.

## Conclusion

We may conclude that the poorer performance of the patients on the capture task is possibly related to impairments of brain structures such as the substantia nigra or the reticular formation. However, as we did not actually measure the eye movements elicited by this task and have no evidence of lesions in substantia nigra or lower brain stem in our subjects, there may be other factors responsible for the observed perceptual abnormalities. In PD, cognitive impairment includes disturbances of language, memory, visuospatial cognition and executive functions. The disturbance of elementary sensory processes may only be part of the impairment in higher-order brain functions that is ultimately responsible for the observed deficits.

The present study indicates that the oculomotor capture task is a sensitive instrument to detect early perceptual deficits in PD. The effect sizes found are such that a clinical relevance of the impairments for the activities of daily living cannot be excluded. In combination with other perceptual impairments involving visual, olfactory, auditive and somatosensory processes, the deficits found in the present study are likely to interfere with daily activities.

Future research using this capture task in still earlier stages of PD, other patient groups and prospective studies in individuals at risk for developing PD will be needed to evaluate whether reduced inhibition of saccades to irrelevant stimuli as determined by this task can be used as an early indicator of the development of PD.

## Competing interests

The author(s) declare that they have no competing interests.

## Authors' contributions

JBD and DS made substantial contributions to conception and design, acquisition, analysis, and interpretation of data and writing the manuscript. HWB, ECW and JT were involved in interpretation of data and in revising the manuscript. All authors read and approved the final manuscript.

## Pre-publication history

The pre-publication history for this paper can be accessed here:



## Figures and Tables

**Table 1:** Subject characteristics

	Patients (n = 11)	Controls (n = 12)
Age (years) ± SD (range)	57.1 ± 9.1 (44–70)	60.6 ± 7.5 (49–77)
Sex (male/female)	6/5	2/10
Education, SOI	6.0 ± 1.3	6.0 ± 1.3
UPDRS III	13.9 ± 7.3	n.a.
Hoehn and Yahr (1/1,5/2/2.5)	7/1/2/1	n.a.
Disease duration (years)	2.3 ± 1.9	n.a.
MMSE	28.8 ± 1.34	28.7 ± 1.0
